# Increased long noncoding RNA maternally expressed gene 3 contributes to podocyte injury induced by high glucose through regulation of mitochondrial fission

**DOI:** 10.1038/s41419-020-03022-7

**Published:** 2020-09-29

**Authors:** Qiongxia Deng, Ruowei Wen, Sirui Liu, Xiaoqiu Chen, Shicong Song, Xuehong Li, Zhongzhen Su, Cheng Wang

**Affiliations:** 1 Department of Medicine, Division of Nephrology, The Fifth Affiliated Hospital Sun Yat-Sen University, Zhuhai, Guangdong 519000 China; 2 Guangdong Provincial Key Laboratory of Biomedical Imaging, The Fifth Affiliated Hospital Sun Yat-Sen University, Zhuhai, Guangdong 519000 China; 3 Department of Ultrasound, The Fifth Affiliated Hospital Sun Yat-Sen University, Zhuhai, Guangdong 519000 China

**Keywords:** Diabetes complications, Glomerular diseases, Diabetes complications

## Abstract

Excessive mitochondrial fission plays a key role in podocyte injury in diabetic kidney disease (DKD), and long noncoding RNAs (lncRNAs) are important in the development and progression of DKD. However, lncRNA regulation of mitochondrial fission in podocytes is poorly understood. Here, we studied lncRNA maternally expressed gene 3 (Meg3) in mitochondrial fission in vivo and in vitro using human podocytes and Meg3 podocyte-specific knockdown mice. Expression of lncRNA Meg3 in STZ-induced diabetic mice was higher, and correlated with the number of podocytes. Excessive mitochondrial fission of podocytes and renal histopathological and physiological parameters were improved in podocyte-specific Meg3 knockdown diabetic mice. Elongated mitochondria with attenuated podocyte damage, as well as mitochondrial translocation of dynamin-related protein 1 (Drp1), were decreased in Meg3 knockout podocytes. By contrast, increased fragmented mitochondria, podocyte injury, and Drp1 expression and phosphorylation were observed in lncRNA Meg3-overexpressing podocytes. Treatment with Mdivi1 significantly blunted more fragmented mitochondria and reduced podocyte injury in lncRNA Meg3-overexpressing podocytes. Finally, fragmented mitochondria and Drp1 mitochondrial translocation induced by high glucose were reduced following treatment with Mdivi1. Our data show that expression of Meg3 in podocytes in both human cells and diabetic mice was higher, which regulates mitochondrial fission and contributes to podocyte injury through increased Drp1 and its translocation to mitochondria.

## Introduction

The prevalence of diabetes mellitus is increasing every year in China, and is currently estimated at 11.6%^1^. Approximately 30–40% of patients develop diabetic kidney disease (DKD)^2^. Most patients eventually progress to renal failure despite the wide use of renin–angiotensin aldosterone system inhibitors, intensive blood glucose, and blood pressure control^3–5^. Therefore, it is urgent to further explore the molecular mechanisms of DKD and to develop new strategies for the prevention and treatment of DKD.

Podocyte damage is the primary pathological change evident in DKD^5^. Loss of >20% of podocytes results in irreversible glomerular damage that has been shown to progress to renal failure in animals^6,7^. Sufficient energy supply from mitochondria is crucial for podocytes to maintain their filter barrier, and insufficient energy due to mitochondrial damage results in the destruction of actin and intermediate filaments, initiating podocyte damage^8,9^. Therefore, exploring the mechanism underlying mitochondrial injury in podocyte damage will help identify therapeutic targets. Mitochondria undergo antagonistic processes of fission and fusion continuously^10,11^. Dynamin-related protein 1 (Drp1) plays a key role in fission, while mitofusins 1 (Mfn1), mitofusins 2 (Mfn2), and optic atrophy1 (Opa1) are responsible for fusion. Excessive mitochondrial fission (fragments) has been shown to contribute to the development and progression of DKD^12^. Wang et al.^13^ provided the first evidence that excessive mitochondrial fission was a core feature of mitochondrial dysfunction in podocytes in DKD.

Long noncoding ribonucleic acids (lncRNAs) can modulate gene expression through a variety of mechanisms, including oxidative stress, inflammatory response, apoptosis, differentiation, and nuclear architecture^14–16^. Among identified lncRNAs, maternally expressed gene 3 (Meg3) has been implicated in the regulation of mitochondrially mediated apoptosis^17^. Nevertheless, few studies have focused on the role of Meg3 in the development of DKD. We hypothesized that Meg3 might contribute to podocyte damage by mediating excessive mitochondrial fission in DKD. To test this hypothesis, we cultured human podocytes and generated podocyte-specific Meg3 knockdown mice to investigate the role of Meg3 in podocyte mitochondrial fission and regulation of DKD progression.

## Results

### lncRNA Meg3 expression is enhanced in human podocytes cultured with high glucose and podocytes of STZ-induced mice

**Fig. 1:**
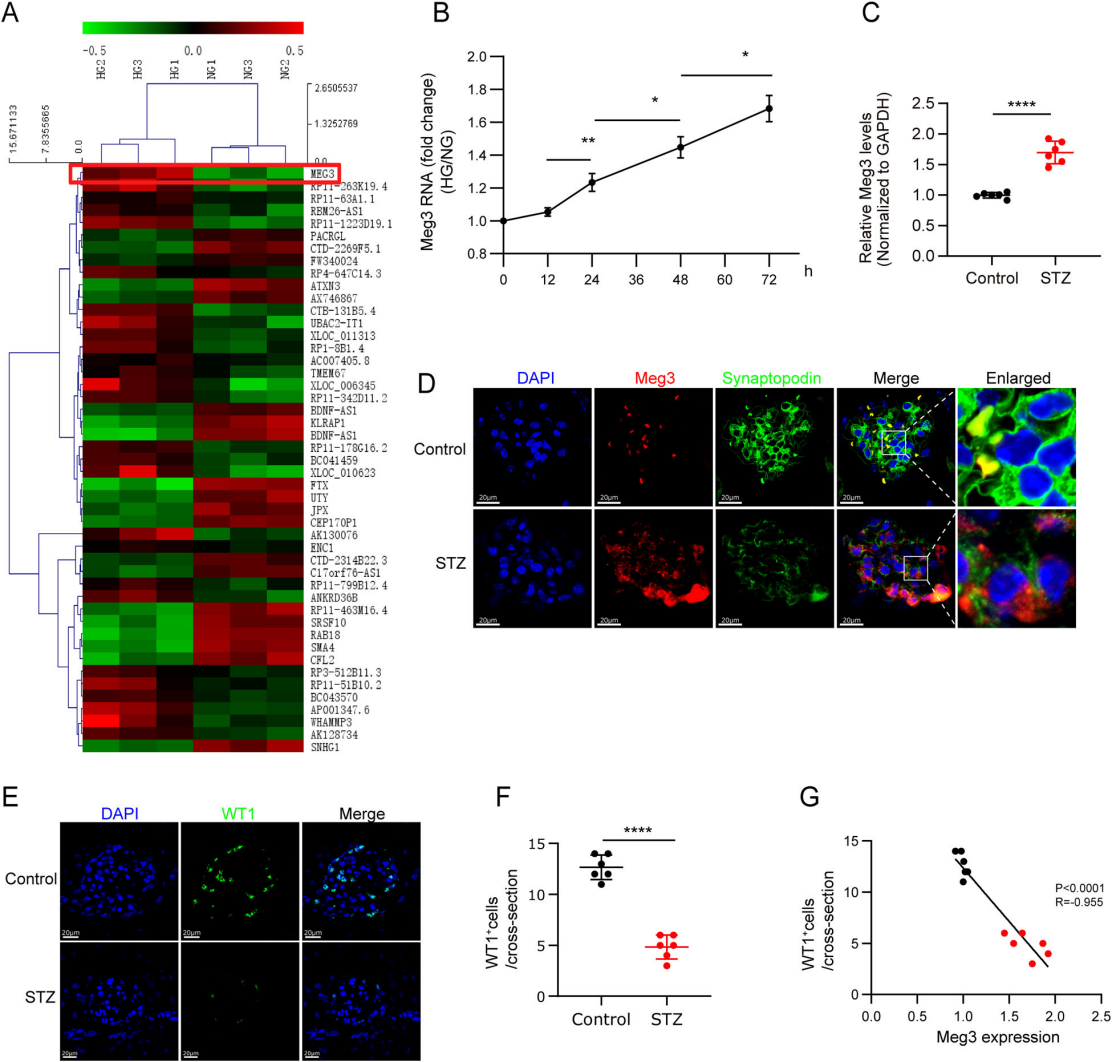
LncRNA Meg3 expression is increased in podocytes in diabetic kidney disease. **a** Heat map of expression of lncRNAs in human podocytes cultured with NG or HG for 72 h. **b** Expression of lncRNA Meg3 in human podocytes was upregulated by HG in a time-dependent manner. **c** Expression of lncRNA Meg3 in podocytes isolated from mice. **d** Confocal FISH + IF images showing localization and expression of lncRNA Meg3 in podocytes of kidney glomeruli from diabetic (STZ-induced) mice compared with glomeruli from non-diabetic (wild-type) control mice (original magnification, ×630, scale bar, 20 μm). **e** Confocal IF images showing WT1-stained kidney cortex sections (original magnification, ×630, scale bar, 20 μm). **f** The number of podocytes per glomerular cross-section was detected using WT1 antibody in 50 randomly selected glomeruli per mouse in each group. **g** Correlation of lncRNA Meg3 with the number of podocytes in a partial correlation analysis. Data were shown as mean ± SD in all statistical graphs (*n* = 6 mice per group), *P < 0.05, **P < 0.01, ****P < 0.0001.

A lncRNA array was used to identify lncRNAs implicated in the regulation of human podocytes with high glucose (HG). The expression of Meg3 was higher in human podocytes treated with HG relative to control (Fig. 1a); this was confirmed by real-time PCR analysis (Fig. 1b). Quantitative real-time PCR (qRT-PCR), fluorescence in situ hybridization (FISH), and immunofluorescence (IF) analyses showed increased expression of Meg3 in podocytes from STZ-induced diabetic mice compared with control mice. Meg3 was primarily localized in the cytoplasm of podocytes (Fig. 1c, d). Furthermore, WT1 IF staining was performed to quantify podocyte number per glomerular cross-section^18^. STZ-induced mice had a significantly decreased number of podocytes per glomerular section compared with control mice, and a negative correlation between the number of podocytes and the expression of Meg3 in podocytes was found (Fig. 1e–g). These data suggest that expression of Meg3 in podocytes is increased and might be involved in podocyte injury in diabetic mice.

### Generation of podocyte-specific lncRNA Meg3 knockdown mice

**Fig. 2:**
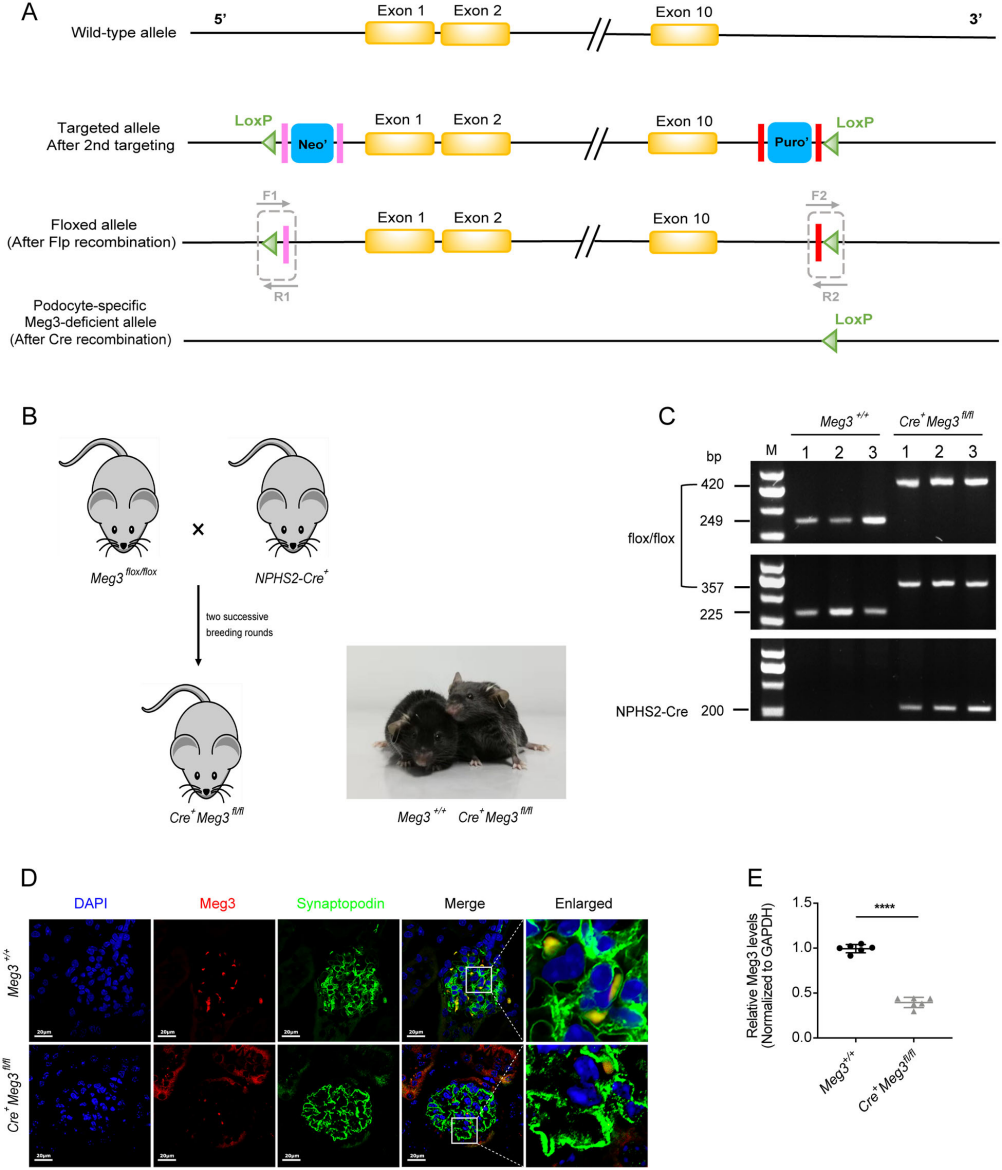
Generation of podocyte-specific knockdown of LncRNA Meg3 mice. **a** Schematic diagram of target strategy of Meg3 deficiency in podocytes using the Cre-loxP system. **b** The breeding strategy to generate podocyte-specific Meg3-deficient mice (*Cre*^*+*^*Meg3*^*fl/fl*^ mice) and the actual appearance of *Meg3*^*+/+*^ mice and *Cre*^*+*^*Meg3*^*fl/fl*^ mice were shown. **c** PCR genotyping of *Meg3*^*+/+*^ mice and *Cre*^*+*^*Meg3*^*fl/fl*^ mice by tail RNA at the age of 3 weeks. **d** Representative images of immunofluorescence (IF) staining for Synaptopodin (green) and fluorescence in situ hybridization (FISH) for Meg3 (red) in glomeruli from *Meg3*^*+/+*^ mice and *Cre*^*+*^*Meg3*^*fl/fl*^ mice (original magnification, ×630, scale bar, 20 μm) (*n* = 6 mice per group). **e** Total RNA was extracted from primary podocytes isolated from *Cre*^*+*^*Meg3*^*fl/fl*^ mice and *Meg3*^*+/+*^ mice, and real-time PCR was performed for Meg3 expression (*n* = 6 mice per group). Data were presented as mean ± SD in all statistical graphs (*n* = 6 mice per group). ****P < 0.0001.

To assess the contribution of lncRNA Meg3 in mitochondrial fission and podocyte damage in vivo, podocyte-specific Meg3 knockdown mice were generated using the Cre-loxP system (Fig. 2a–c). To further confirm conditional knockdown of Meg3 in podocytes, FISH, IF, and qRT-PCR analyses were performed. Meg3 expression was decreased in podocytes of *Cre*^*+*^*Meg3*^*fl/fl*^ mice relative to *Meg3*^*+/+*^ mice (Fig. 2d, e), while no difference on Meg3 expression was found in tubular cells, liver, heart, and spleen between *Cre*^*+*^*Meg3*^*fl/fl*^ and *Meg3*^*+/+*^ mice (Supplementary Fig. 1).

### Knockdown of lncRNA Meg3 in podocytes ameliorated albuminuria and renal dysfunction of STZ-induced diabetic mice

**Fig. 3:**
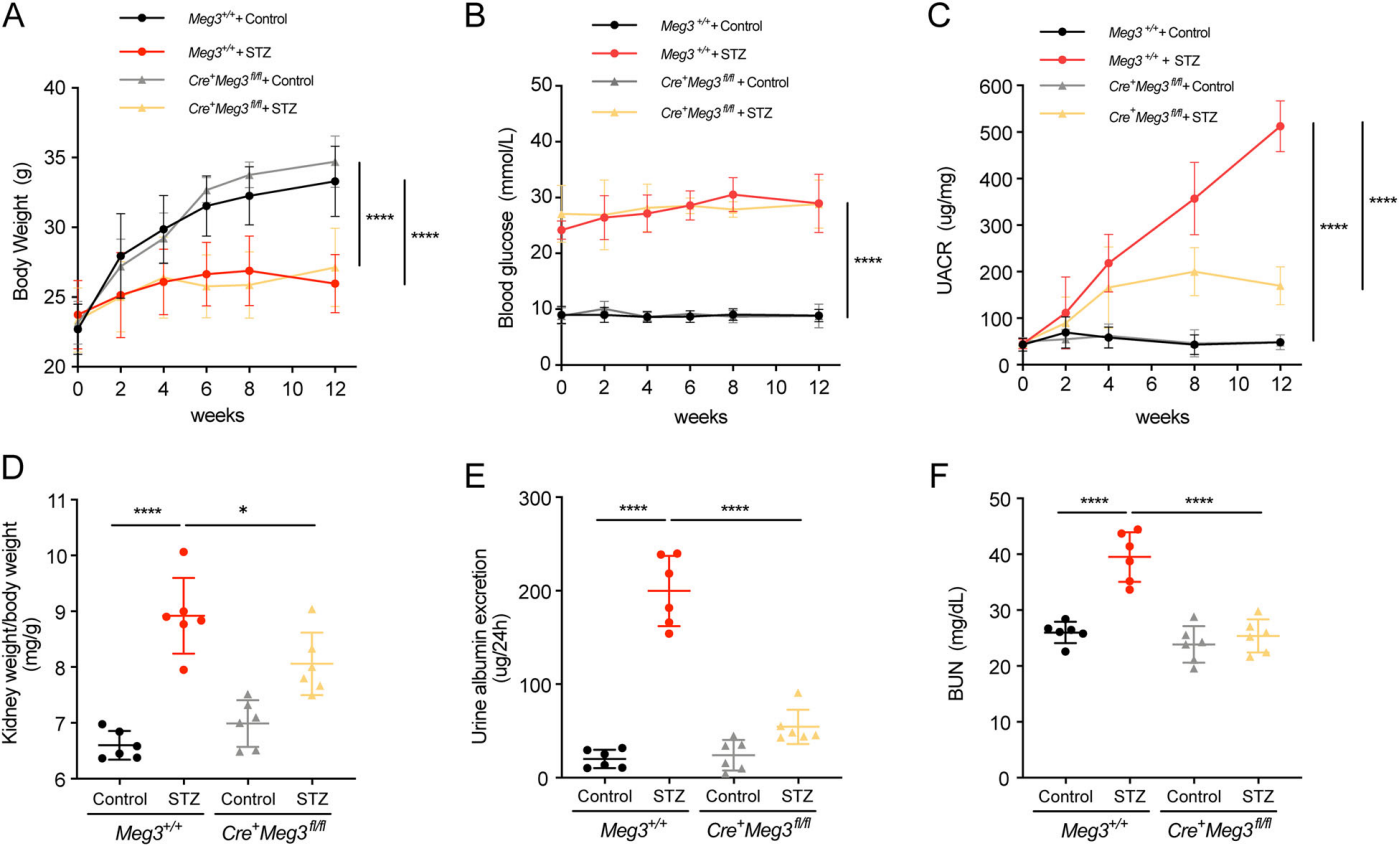
Knockdown of lncRNA Meg3 in podocytes ameliorated albuminuria and renal dysfunction in STZ-induced diabetic mice. **a** Changes of body weight measured every 2 weeks during the 12-week follow-up period. **b** Changes of blood glucose measured every 2 weeks during the 12-week follow-up period. **c** Changes of urinary urine albumin/creatinine ratio (UACR) in each group during the 12-week follow-up period. **d** Changes of kidney weight/body weight ratio in each group. **e** Changes of urine albumin excretion at the time of 12 weeks after the establishment of hyperglycemia. **f** Changes of blood urea nitrogen (BUN) in each group. Data were shown as mean ± SD in all statistical graphs (*n* = 6 mice per group). *P < 0.05 and ****P < 0.0001.

To determine the effects of podocyte-specific Meg3 deficiency under diabetic conditions, we generated a diabetic state by intraperitoneal injection of STZ into *Meg3*^*+/+*^ and *Cre*^*+*^*Meg3*^*fl/fl*^ mice. Diabetic mice exhibited lower body weights and higher blood glucose, but no significant differences were observed between the two groups (Fig. 3a, b). Higher UACR, kidney/body weight ratio, and increased levels of urine albumin excretion (UAE) and serum blood urea nitrogen were detected in diabetic mice; all of these parameters in diabetic *Cre*^*+*^*Meg3*^*fl/fl*^ mice were significantly lower than in diabetic *Meg3*^*+/+*^ mice (Fig. 3c–f). These data suggest that Meg3-specific knockdown in podocytes can improve physiological parameters independent of blood glucose in diabetic mice.

### Knockdown of Meg3 in podocytes improved glomerular injury and podocyte mitochondrial fission in STZ-induced diabetic mice

**Fig. 4:**
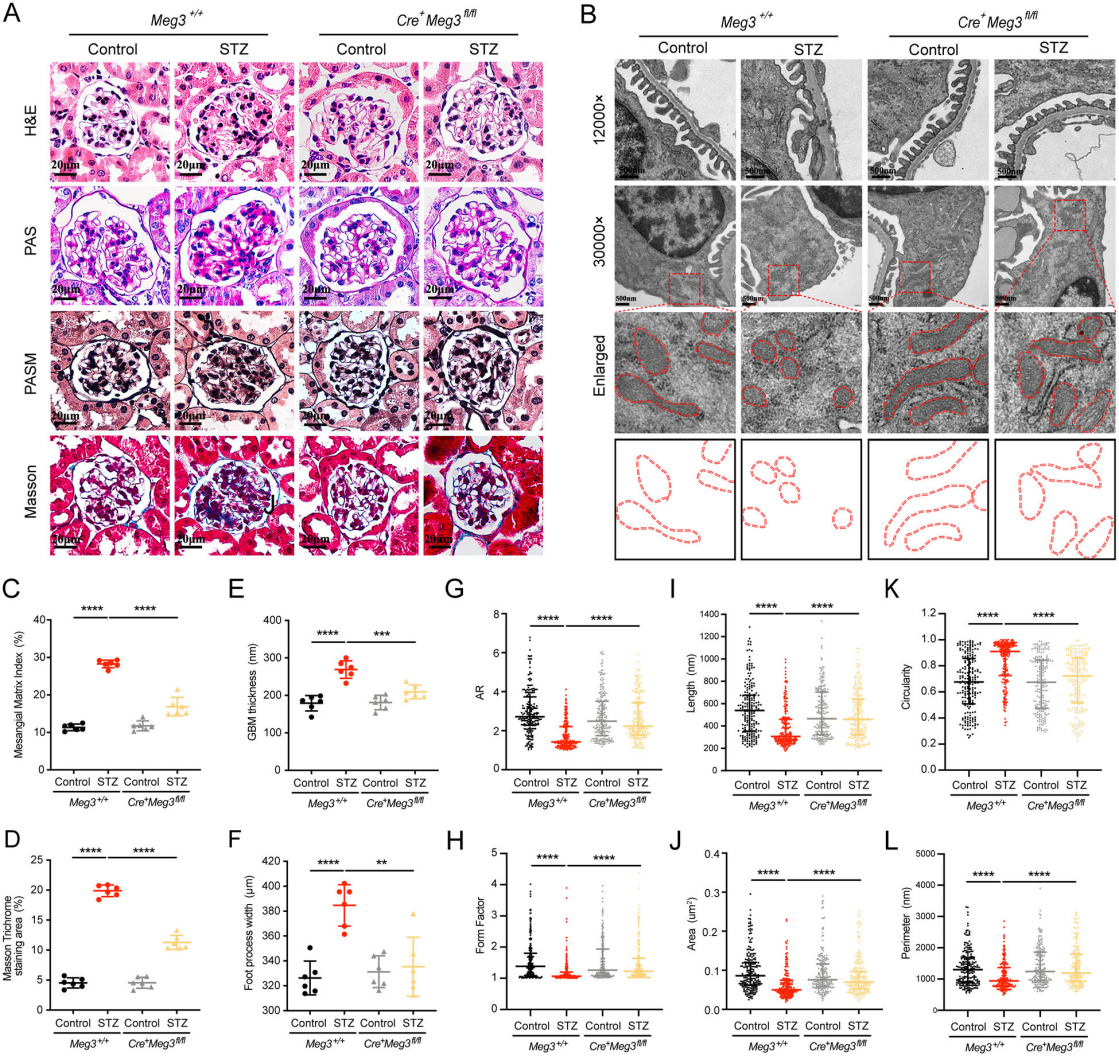
Glomerular injury and podocyte excessive mitochondria fusion were attenuated in STZ-induced diabetic mice with podocyte-specific lncRNA Meg3 knockdown. **a** Representative images of HE, periodic Acid-Schiff (PAS), PASM, and Masson staining in kidney sections (original magnification, ×400; scale bar, 20 μm). **b** Representative images of transmission electron microscopy (TEM) visualizing the glomerular ultrastructure (top panel, original magnification, ×12,000). Representative TEM images in lower panels display mitochondrial shape in podocytes (original magnification, ×30,000). The red dotted lines indicated the mitochondria, with a double membrane. **c** Comparison of mesangial matrix index in each group. **d** Comparison of Masson Trichrome staining area in each group. **e** Comparison of glomerular basement membrane (GBM) thickness in each group. **f** Degree of podocyte foot process effacement was quantified by assessing foot process width using TEM. **g** Comparison of mitochondria aspect ratio (AR) in podocytes of different group. **h** Comparison of mitochondria form factor in podocytes of different group. **i** Comparison of mitochondrial length in podocytes of a different group. **j** Comparison of the mitochondrial area in podocytes of a different group. **k** Comparison of mitochondrial circularity in podocytes of a different group. **l** Comparison of the mitochondrial perimeter in podocytes of a different group. Two hundred mitochondria were randomly selected in each group when mitochondrial morphology analyses were done. Normally distributed data were shown as mean ± SD, and non-normally distributed data were shown as median (interquartile range) in all statistical graphs (*n* = 6 mice per group). **P < 0.01, ***P < 0.001, and ****P < 0.0001.

To ascertain the influence of Meg3 knockdown in podocytes on renal pathology under diabetic conditions, histopathological and morphometric analyses were performed. Diabetic mice exhibited more severe mesangial matrix expansion and glomerulosclerosis, increased glomerular basement membrane thickness, and aggravated podocyte foot process effacement, and these pathological features were improved in diabetic *Cre*^*+*^*Meg3*^*fl/fl*^ mice (Fig. 4a, c–f). Using transmission electron microscopy (TEM), more fragmented mitochondria were observed in podocytes from diabetic states, demonstrated by the lower aspect ratio (AR) and form factor of podocyte mitochondria, while more elongated mitochondria with a higher AR and form factor were found in podocytes from diabetic *Cre*^*+*^*Meg3*^*fl/fl*^ mice relative to diabetic *Meg3*^*+/+*^ mice (Fig. 4b, g–l). These data indicate that Meg3-specific knockdown in podocytes can improve renal pathology and mitochondrial fission in diabetic mice.

### Knockout of Meg3 attenuated HG-induced mitochondrial fission and cell injury in human podocytes

**Fig. 5:**
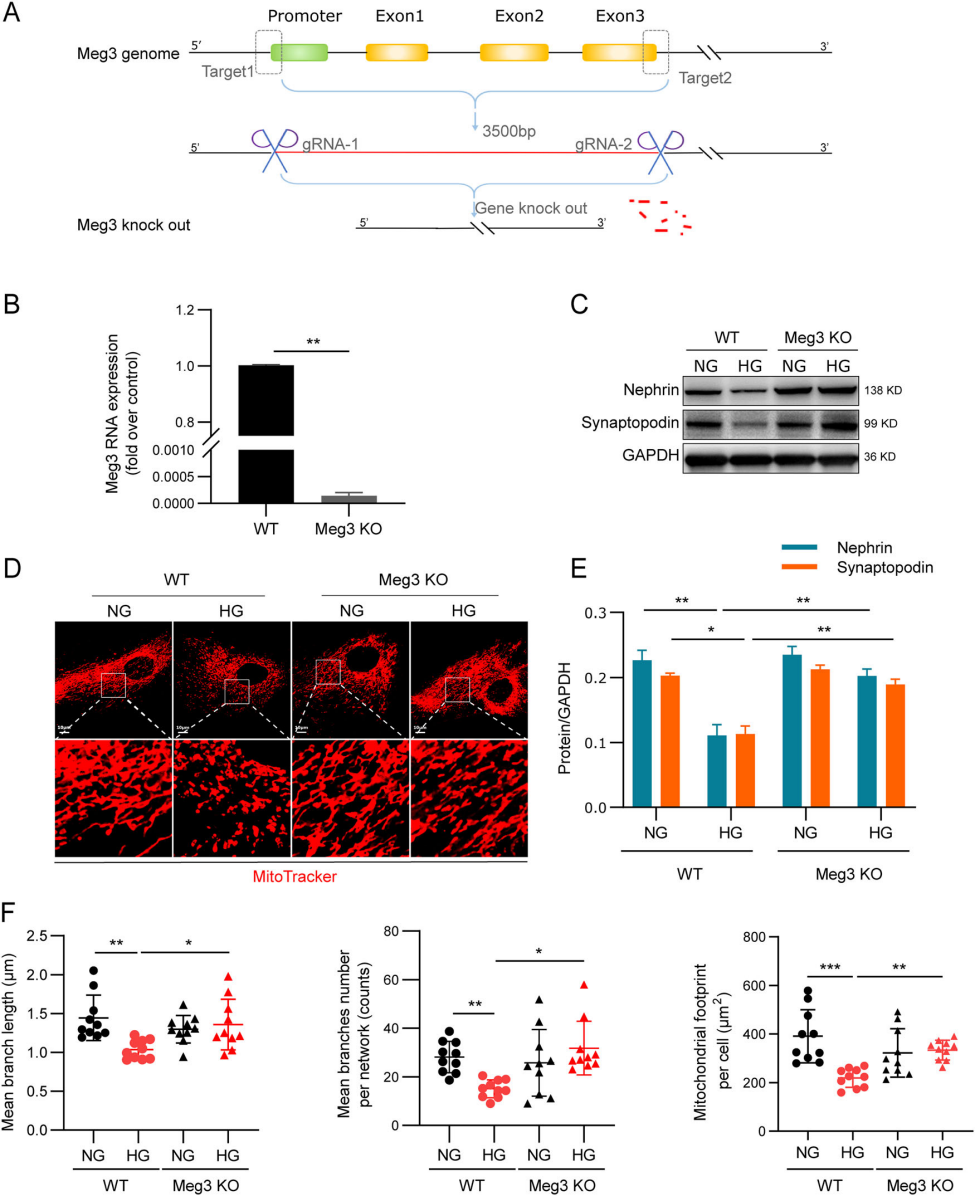
Knockout of lncRNA Meg3 attenuates high glucose-induced mitochondrial fission and cell injury in human podocytes. **a** Schematic diagram of target strategy of Meg3 knockout in human podocytes using the double-nickase/CRISPR-Cas9 System. Two pairs guide RNAs targeted to the promoter region and exon 3 of lncRNA Meg3, and the target sequence is approximately 3500 bp. **b** Expression of lncRNA Meg3 in human podocytes of different groups. **c** Expression of Nephrin and Synaptopodin in human podocytes of different groups. **d** Representative structured of mitochondria of human podocytes were stained by MitoTracker Deep Red and imaged by laser scanning confocal microscopy (original magnification, ×630; scale bar, 10 μm). **e** Quantitative analyses of expression of Nephrin and Synaptopodin in human podocytes of different groups. **f** Quantitative analyses of mitochondrial organization in human podocytes of different groups (mean branch length, mean number of branches per network, and mitochondrial footprint, *n* = 10 cells per group). Data were shown as mean ± SD in all statistical graphs. *P < 0.05, **P < 0.01, and ***P < 0.001 (*n* = 6 per group).

To determine the role of Meg3 in mitochondrial fission and cell injury in podocytes induced by HG, we generated a stable human podocyte cell line for knockout (KO) of Meg3 (Supplementary Fig. 2A–D and Fig. 5a, b). Consistent with our previous report^19,20^, cultured human podocytes were injured by HG, as demonstrated by lower expression of Nephrin and Synaptopodin (Fig. 5c, e). At the same time, highly fragmented and discrete mitochondria were observed in podocytes incubated with HG (Fig. 5d, f), while podocyte injury was attenuated in the absence of Meg3. Moreover, less fragmented, more elongated, and interconnected mitochondria were observed in Meg3 KO podocytes. The trend was the same in the detection of mitochondrial membrane potential and ATP (Supplementary Fig. 2E–G). These data indicate that downregulation of Meg3 might protect against excessive mitochondrial fission and podocyte injury in HG conditions.

### Overexpression of Meg3 aggravated HG-induced mitochondrial fission and cell injury in human podocytes

**Fig. 6:**
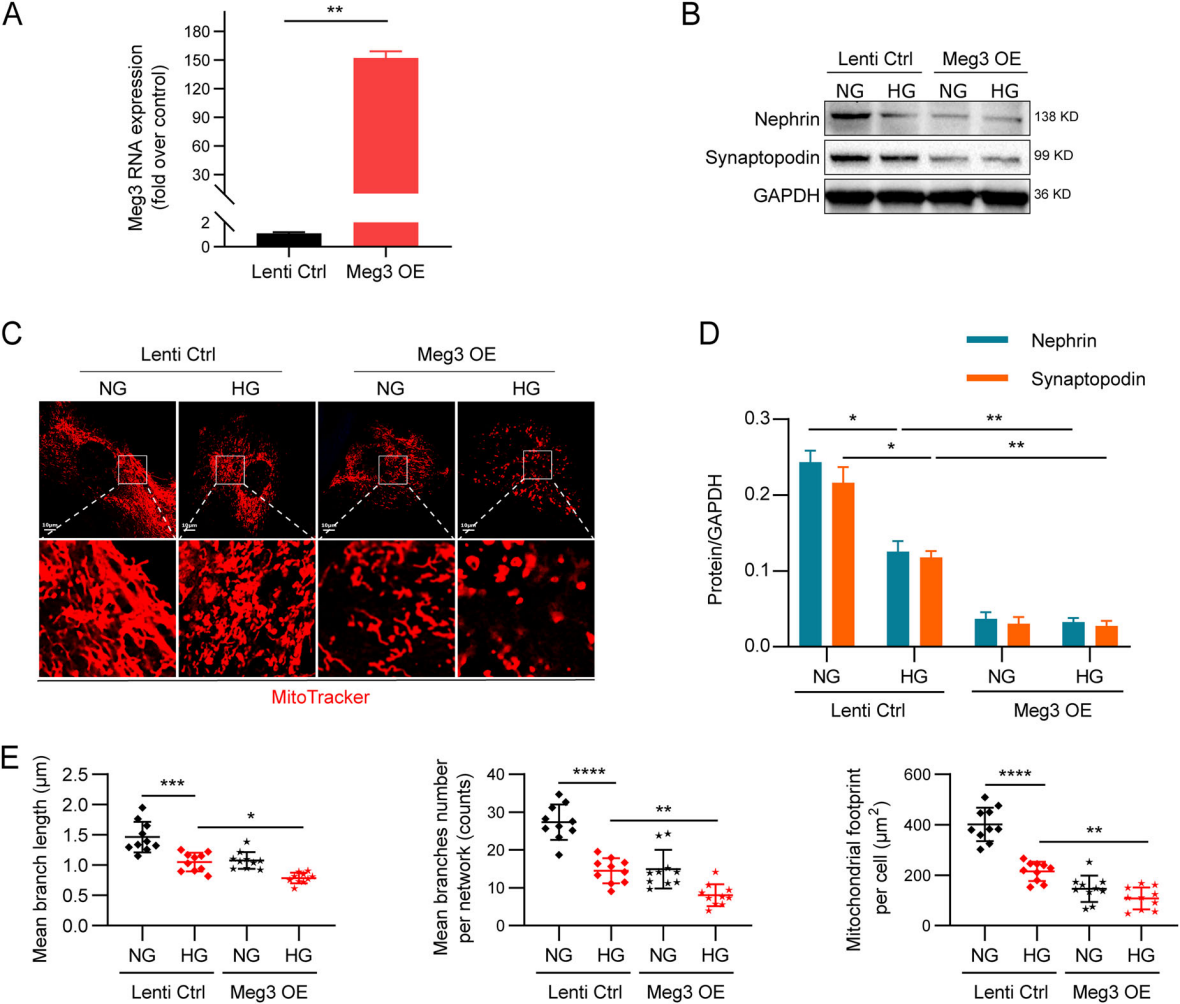
Overexpression of lncRNA Meg3 aggravated high glucose-induced mitochondrial fission and cell injuries in human podocytes. **a** Expression of lncRNA Meg3 in Meg3 overexpression podocytes compared with the control. **b** Expression of Nephrin and Synaptopodin in human podocytes of different groups. **c** Representative structured of mitochondria of human podocytes were stained by MitoTracker Deep Red and imaged by laser scanning confocal microscopy (original magnification, ×630; scale bar, 10 μm). **d** Quantitative analyses of expression of Nephrin and Synaptopodin in human podocytes of different groups. **e** Quantitative analyses of mitochondrial organization on human podocytes from different groups (mean branch length, mean number of branches per network and mitochondrial footprint, *n* = 10 cells per group). Data were shown as mean ± SD in all statistical graphs. *P < 0.05, **P < 0.01, ***P < 0.001, and ****P < 0.0001 (*n* = 6 per group).

To further confirm the role of Meg3 in mitochondrial fission and podocyte damage, we generated Meg3-overexpressing (OE) podocytes by infection with lentivirus-expressing Meg3 (Supplementary Fig. 3A–C and Fig. 6a). lncRNA Meg3 OE cells were damaged more severely compared with control cells under HG or normal glucose (NG) conditions, as demonstrated by decreased expression of Nephrin and Synaptopodin (Fig. 6b, d). In addition, more highly fragmented and discrete mitochondria were found in Meg3 OE cells, including significantly lower branch lengths, network sizes, and footprints (Fig. 6c, e). The generation of ATP was also dramatically reduced in lncRNA Meg3 OE cells compared with WT podocytes incubated in HG (Supplementary Fig. 3D). Collectively, these results suggest the possible roles of higher lncRNA Meg3 expression in the induction of mitochondrial fission and podocyte injury in HG.

### lncRNA Meg3 promotes mitochondrial fission in podocytes by regulation of Drp1 and its translocation to mitochondria

**Fig. 7:**
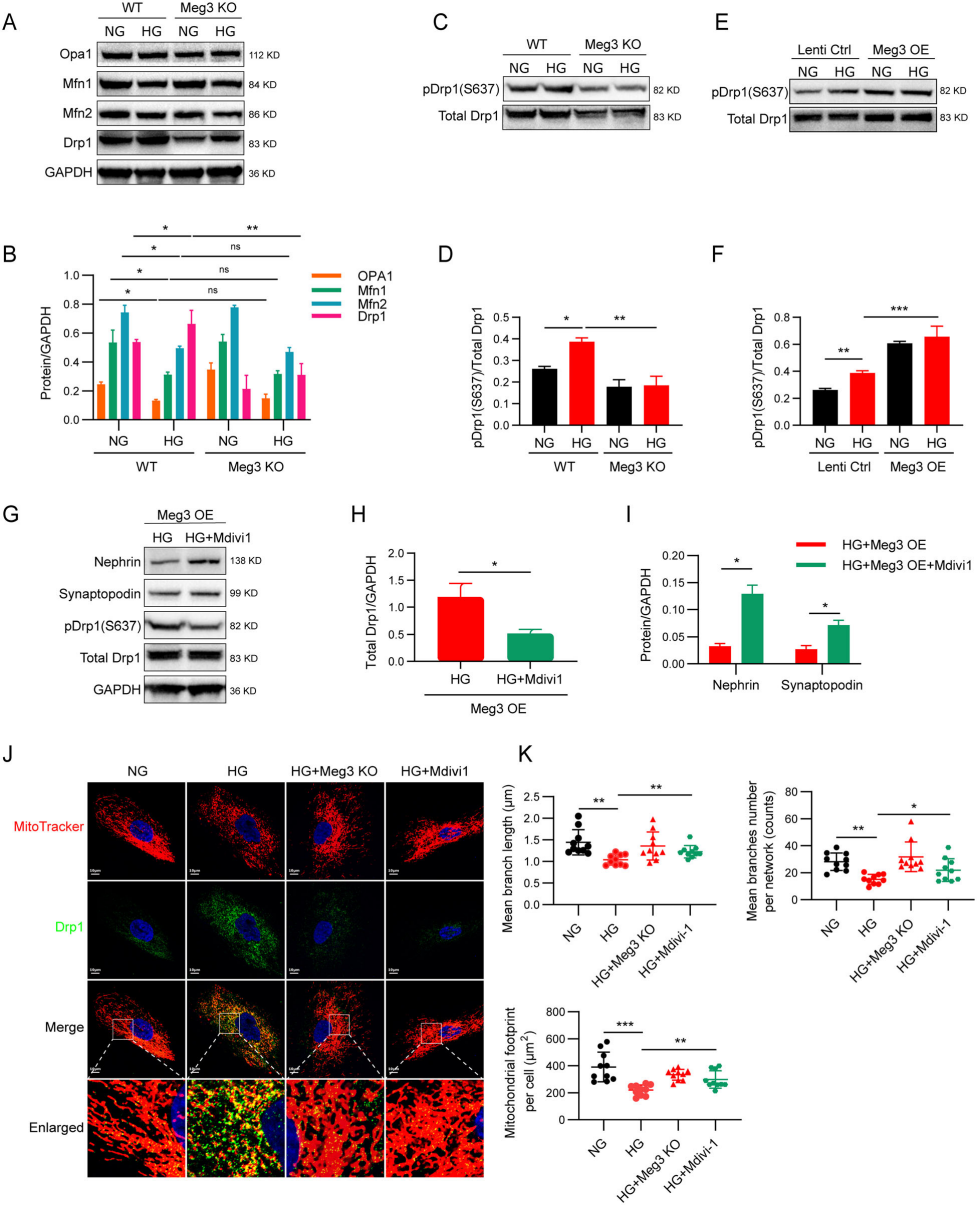
LncRNA Meg3 promotes fission of the mitochondria in podocytes by regulation of Drp1 and its translocation to mitochondria. **a**, **b** Western blot analysis of proteins (Opa1, Mfn1, Mfn2 and Drp1) involved in the regulation of mitochondrial fission and fusion in podocytes from different groups. **c**–**f** Western blot analysis of pDrp1 (Ser637) in Meg3 KO and OE podocytes. **g**–**i** Western blot analysis of proteins in podocytes treated with HG or HG + Mdivi1 in Meg3 OE cell line. **j** Representative confocal images of mitochondria in human podocytes were stained by MitoTracker Deep Red and incubated with anti-Drp1 (original magnification, ×630; scale bar, 10 μm). **k** Quantitative analyses of mitochondrial organization on human podocytes from different groups (mean branch length, mean number of branches per network, and mitochondrial footprint, *n* = 10 cells per group). Data were shown as mean ± SD in all statistical graphs. *P < 0.05, **P < 0.01, ***P < 0.001 (*n* = 6 per group), and n.s., not significant.

We then assessed the role of Meg3 in mitochondrial fission in podocytes in diabetic states. First, we explored the changes in expression of proteins involved in fission and fusion of mitochondria in wild-type (WT) and Meg3 KO human podocytes cultured under NG or HG conditions. The expression of Opa1, Mfn1, and Mfn2 in podocytes cultured with HG was reduced relative to NG; however, no differences in the expression levels of these proteins were found in lncRNA Meg3 KO cells relative to WT podocytes (Fig. 7a, b). Drp1 expression in WT podocytes cultured with HG was higher than in podocytes treated with NG, while the expression of Drp1 in Meg3 KO cells cultured in HG was greatly reduced compared with WT podocytes cultured with HG. IF staining showed that Drp1 translocated to mitochondria in podocytes cultured with HG; this mitochondrial translocation was blocked in Meg3 KO podocytes (Fig. 7j). These data suggest that Drp1 might be related to Meg3-mediated mitochondrial fission and podocyte injury.

Several kinases can influence the subcellular localization of Drp1 by phosphorylating two conserved serine residues of Drp1^21^. Increased phosphorylation of Drp1 at Ser600 (Ser637 in humans) is essential for HG-mediated mitochondrial fission^22^. Thus, we detected phosphorylation of Ser637 in Meg3 KO and OE podocytes. Increased phosphorylation of Ser637 of Drp1 was observed in human podocytes cultured in HG relative to podocytes incubated in NG, while the phosphorylation induced by HG was reduced in Meg3 KO podocytes. By contrast, phosphorylation was increased in Meg3 OE podocytes (Fig. 7c–f). Mdivi1, a pharmacological inhibitor of Drp1, blocks this phosphorylation and subsequent mitochondrial translocation^22^. Human podocyte injury was attenuated following treatment with Mdivi1, as demonstrated by the increased expression of Nephrin and Synaptopodin in podocytes treated with Mdivi1 relative to those untreated in HG conditions (Fig. 7g–i). In addition, Mdivi1 treatment blocked Drp1 translocation to mitochondria and improved mitochondrial morphology: less fragmented, more elongated, and interconnected mitochondria were observed compared with lncRNA Meg3 OE podocytes cultured in HG (Fig. 7j, k). These data suggest that Meg3 can promote mitochondrial fission and induce podocyte injury by regulating Drp1 and its translocation to mitochondria.

### Specific knockdown of LncRNA Meg3 in podocytes alleviated Drp1 and its phosphorylation of Ser600 in podocyte from in STZ-induced diabetic mice

**Fig. 8:**
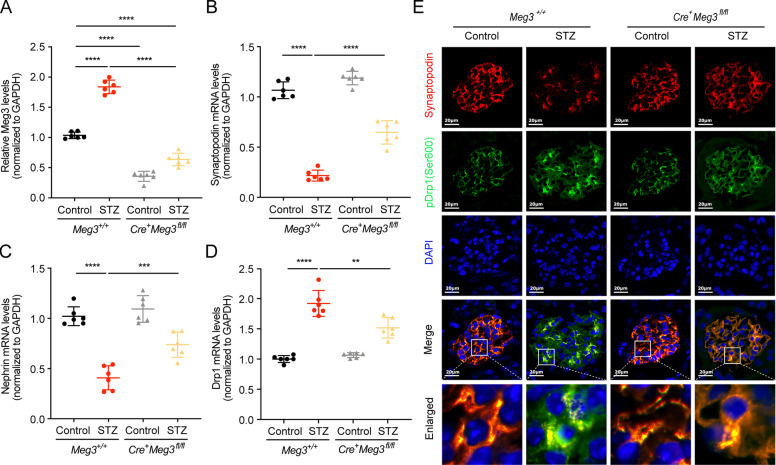
Specific knockdown of lncRNA Meg3 in podocytes alleviated Drp1 and its phosphorylation of Ser600 in podocyte from in STZ-induced diabetic mice. **a** LncRNA Meg3 expression in primary podocytes isolated from mice kidneys. **b** Synaptopodin mRNA expression in primary podocytes isolated from mice kidneys. **c** Nephrin mRNA expression in primary podocytes isolated from mice kidneys. **d** Drp1 mRNA expression in primary podocytes isolated from mice kidneys. **e** Immunofluorescence staining for pDrp1 (Ser600) (green) and Synaptopodin (red) in the paraffin-embedded kidney sections was performed (original magnification, ×630, scale bar, 20 μm). Data were shown as mean ± SD in all statistical graphs (*n* = 6 mice per group). **P < 0.01, ***P < 0.001, and ****P < 0.0001.

Primary mice podocytes were isolated for RNA extraction. Meg3 expression in podocytes from *Cre*^*+*^*Meg3*^*fl/fl*^ mice was lower than those isolated from *Meg3*^*+/+*^ mice (Fig. 8a). mRNA expression of Nephrin and Synaptopodin was also decreased in podocytes from STZ-induced diabetic *Meg3*^*+/+*^ mice compared with control *Meg3*^*+/+*^ mice, while expression of Nephrin and Synaptopodin in diabetic *Cre*^*+*^*Meg3*^*fl/fl*^ mice was higher than in diabetic *Meg3*^*+/+*^ mice (Fig. 8b, c). We also found that the expression of Drp1 mRNA was increased in the diabetic group, and downregulated with Meg3 knockdown (Fig. 8d). Higher expression of pDrp1 (Ser600, corresponding to Ser637 in human Drp1) colocalized with Synaptopodin was observed in glomeruli of diabetic *Meg3*^*+/+*^ mice compared with control *Meg3*^*+/+*^ mice, while these two colocalized expression in diabetic *Cre*^*+*^*Meg3*^*fl/fl*^ mice were lower than diabetic *Meg3*^*+/+*^ mice (Fig. 8e). These data suggest that knockdown of Meg3 in podocytes from STZ-induced diabetic mice can alleviate podocyte injury and reduce Drp1 expression and phosphorylation of Ser600 in podocytes in vivo.

## Discussion

This study identified novel correlations between lncRNA Meg3 expression and podocyte injury, and showed the importance of Meg3 in mediating excessive mitochondrial fission in the development of DKD. First, the increased expression of Meg3 in podocytes from STZ-induced diabetic mice was shown to be primarily localized in the cytoplasm, and was negatively correlated with the number of podocytes. Second, excessive mitochondrial fission of podocytes, as well as key pathological features and biochemistry parameters, were improved in podocyte-specific Meg3 knockdown mice. Third, more fragmented mitochondria, podocyte injury, and increased Drp1 expression and phosphorylation, were observed in podocytes with Meg3 overexpression. By contrast, more elongated mitochondria with attenuated podocyte damage, and decreased Drp1 expression and mitochondrial translocation were observed in podocytes without Meg3. Fourth, pharmacological inhibition of Drp1 significantly blunted increased fragmented mitochondria and reduced podocyte injury in podocytes with Meg3 overexpression. Finally, pharmacological inhibition of Drp1 reduced its mitochondrial translocation, and alleviated the generation of mitochondrial fragments and podocyte damage induced by HG. These findings provide a clear link between Meg3 expression and mitochondrial fission in podocyte injury via increased Drp1 and its recruitment to mitochondria.

Recent evidence has suggested that lncRNAs are centrally involved in the development and progression of DKD. Long et al.^23^ reported that lncRNA taurine-upregulated 1 (Tug1) contributed to the development of DKD, and showed that overexpression of Tug1 could modify the bioenergetics of mitochondria in podocytes of diabetic mice. Bai et al.^24^ reported that lncRNA LINC01619 served as a competing endogenous RNA and regulated endoplasmic reticulum stress in DKD. One report showed that lower lncRNA Meg3 expression was found in renal tissue rather than podocytes from STZ-induced diabetic rat and human DKD, mouse podocytes cultured with HG. We cannot confirm the changes in Meg3 expression in podocytes based on these results considering the different species and experimental conditions^25^. Here, we cultured human podocytes with HG for different amounts of time and isolated podocytes from STZ-induced diabetic mice. This is the first study to assess the expression of Meg3 in human podocytes and primary podocytes isolated from diabetic mice. We observed increased Meg3 expression in these podocytes using different methods. Knockout or knockdown of Meg3 attenuated podocyte damage and improved fragmented mitochondria in podocytes. In addition, overexpression of Meg3 could aggravate HG-mediated mitochondrial fission and cell injury in human podocytes. These results suggest that increased levels of Meg3 play a role in podocyte damage and regulation of mitochondrial fission in diabetic states.

Increasing evidence has shown that abnormal mitochondrial dynamics, defined as an imbalance between mitochondrial fission and fusion, are linked to a number of diseases, including neurodegenerative diseases, cardiovascular diseases, and DKD. Therefore, maintaining the balance between mitochondrial fusion and fission is key to homeostasis^26^. Excessive mitochondrial fission (fragmented mitochondria) is not only a marker of mitochondrial injury, but also a key mechanism of cell injury, which can promote mitochondrial membrane rupture, accelerate mitochondrial damage, and cell death^27,28^. Inhibition of mitochondrial fragmentation could reduce mitochondrial oxidative stress and cell damage^29^. Wang et al.^13^ showed that Drp1 and its Ser637 (mouse Ser600) phosphorylation were required for mitochondrial fission in podocytes. Inhibition of Drp1 in podocytes also significantly blunted mitochondrial fission and improved key pathologic features of DKD^22^. However, there has been no report on lncRNA regulation of mitochondrial fission in podocytes. Here, we studied the lncRNA Meg3 and its role in mitochondrial fission in podocytes in diabetic states. We observed increased Drp1 expression in podocytes cultured with HG and decreased expression of Drp1 in Meg3 KO cells. These data suggest that Drp1 may be important for Meg3-mediated mitochondrial fission and podocyte injury.

Drp1 is primarily localized in the cytosol of mammalian cells and shuttles between the cytosol and mitochondrial surface. Translocation of Drp1 from the cytosol to mitochondria is a key step in mitochondrial fission^30–33^. Normally, Mdivi1, a pharmacological inhibitor of Drp1, is sufficient to block phosphorylation and mitochondrial translocation as Mdivi1 acts at the earliest stages of Drp1 oligomerization^13^. In the present study, we found that Drp1 expression, phosphorylation, and translocation were increased in podocytes in the presence of HG. However, knockout of Meg3 could attenuate increased Drp1 expression and phosphorylation, and reduce mitochondrial translocation of Drp1 induced by HG. By contrast, overexpression of Meg3 increased Drp1 expression and its phosphorylation. Mdivi1 improved podocyte injury induced by Meg3 overexpression, which also blocked Drp1 translocation to mitochondria and improved mitochondrial fission induced by HG. These data suggest that Meg3 can promote mitochondrial fission and induce podocyte injury by increased Drp1 expression and translocation.

Several limitations of the currently presented experiments warrant emphasis: first, no human DKD renal biopsies were investigated. Second, we did not observe mitochondrial morphology and Drp1 translocation to mitochondria due to fluorescence in Meg3 OE podocytes. Third, further study is needed to determine how Meg3 regulates Drp1 and its phosphorylation.

In summary, our data showed increased expression of Meg3 in podocytes from human cells and diabetic mice, which regulates mitochondrial fission and contributes to podocyte injury through increased Drp1 phosphorylation and translocation.

## Materials and methods

### Cell culture

Conditionally immortalized human podocytes, a gift from Prof. Saleem, were cultured as previously described^34^. Podocytes were prepared for experiments with NG (5.5 mmol/l) and high glucose (HG, 30 mmol/l) medium for 72 h. Podocytes were pretreated with 20 μM Mdivi1 (Meilune, MB4708; a selective Drp1 inhibitor) for 4 h to investigate the role of Drp1.

### Generation of Meg3 podocyte-specific knockdown mice

C57BL/6N mice with Meg3 targeting vector were generated as shown in Fig. 2. Heterozygotes (*Meg3*^*flox/+*^) were confirmed as germline transmitted via crossbreeding F0 founder mice with Flp deleter. Homozygotes (*Meg3*^*flox/flox*^) were generated by mating *Meg3*^*flox/+*^ mice with each other. *Meg3*^*flox/flox*^ mice were further crossed with podocin promoter (NPHS2)-driven Cre recombinase transgenic mice (NPHS2-Cre) in two successive breeding rounds to generate homozygous podocyte-specific Meg3-deficient mice. Male offspring carrying NPHS2-Cre and the floxed alleles of Meg3 (*NPHS2-Cre*^*+*^ *Meg3*^*flox/flox*^ mice, referred to as *Cre*^*+*^*Meg3*^*fl/fl*^) were chosen as the experimental group (*n* = 12), while Cre recombinase-negative littermate offspring with two wild-type alleles (*Meg3*^*+/+*^) were used as the control group (*n* = 12). All mice were maintained on a normal chow diet and housed at 22–26 °C in a quiet room with a 12 h light/dark cycle.

### STZ-induced diabetic murine model

*NPHS2-Cre*^*+*^ *Meg3*^*flox/flox*^ and *Meg3*^*+/+*^ mice were randomly divided into diabetic and non-diabetic groups, respectively. All the following animal experiments were evaluated in a blinded manner. In the diabetic group, 8-week-old mice were administered streptozotocin (S0130, Sigma-Aldrich, USA) in 0.1 mol/l citrate buffer (pH 4.5) by intraperitoneal injection after a 12 h fast for five consecutive days (50 mg/kg/day). Mice in the non-diabetic group were injected with citrate buffer. One week after the last injection, blood glucose levels of the mice were determined, and mice with fasting blood glucose levels >200 mg/dl were confirmed to be diabetic^35^. All animal experiments were performed with the approval of the Institutional Animal Care and Use Committee of our hospital.

### Arraystar lncRNA array

The assays were performed to explore the potential lncRNAs involved in DKD and the details could be found in Supplementary methods.

### CRISPR/Cas9-mediated targeting

The double-nickase/CRISPR-Cas9 System was used to generate stable clones with KO of lncRNA Meg3 in cultured human podocytes, and the details could be found in supplementary methods.

### Lentiviral transduction

A stable lncRNA Meg3 OE human podocyte line was established using lentivirus infection, and the details could be found in Supplementary methods.

### Mitochondrial morphology assessment

Human podocytes were incubated with 250 nM MitoTracker Deep Red (M22426, Invitrogen, Carlsbad, CA, USA) for 30 min, followed by the addition of mountant and DAPI (4′,6-diamidino-2-phenylindole; P36941; Thermo Fisher Scientific, Waltham, MA, USA). Images were taken from randomly selected fields of view using confocal microscopy (LSM880, Carl Zeiss, Germany), and mitochondrial morphology was assessed using the semiautomated morphometric tool MiNA within Fiji^36,37^. A minimum of ten cells were analyzed per condition in a total of these three parameters.

### Western blot analysis and qRT-PCR

Details could be found in Supplementary methods.

### FISH and IF

Fresh tissue was fixed with general purpose tissue fixer (G1101, Servicebio, China) for 1–2 h. The dehydrated tissue was buried using OCT (4583, Sakura, USA), and sliced with CRYOSTAR NX50 (Thermo Fisher Scientific). Following repair in citrate buffer under repair conditions, the sections were digested with 20 μg/ml protease K (G1205, Servicebio) at 37 °C for 5 min. The samples were hybridized overnight in 8 ng/μl m-Meg3 probe (5′-CY3-CCTCCATTTGCCTCATAATCCAATCAGCCCCT-CY3-3′) hybridization solution at 37 °C prior to incubation with anti-Synaptopodin (sc-515842, 1:100; Santa Cruz Biotechnology, Santa Cruz, CA, USA) overnight at 4 °C. Sections were then incubated with FITC-labeled secondary antibody at room temperature for 1 h. Nuclei were counterstained with DAPI (Servicebio) for 5 min. Finally, anti-fluorescence quenching tablets were added to seal the slices. All microscopic images were recorded using confocal microscopy (LSM880, Carl Zeiss).

### Isolation of primary mouse podocytes

Mice kidneys were removed and cut into pieces on ice, digested in medium containing collagenase A (Sigma-Aldrich) and dispase (Roche, Switzerland) at 37 °C, and passed through 100 and 70 µm sieves in succession. The filtered cells were co-incubated with biotinylated-podocalyxin antibody and biotinylated-Kirrel2 (Neph3) antibody (R&D, USA), and detached using streptavidin M280 magnetic Dynabeads (Invitrogen). The magnetically attracted cells were considered primary podocytes and processed for RNA or protein preparation^23,38^.

### Urine and serum analysis

Details could be found in Supplementary methods

### Histopathology analysis by light microscope and TEM

Details could be found in Supplementary methods.

### Statistical analyses

Data are expressed as mean ± standard deviation, and all experiments were done at least three independent times, unless stated otherwise. Data were evaluated with GraphPad Prism (version 8.4, GraphPad Software, La Jolla, CA, USA) using two-tailed multivariate analysis of variance for repeated measures for comparisons of multiple groups and Student’s *t* test for comparisons between two groups, and non-normal distributed data were performed by Mann–Whitney test. The differences were considered significant at *P* < 0.05.

## Supplementary information

Supplementary methods

Fig-1S

Fig-2S

Fig-3S

Supplementary Table S1

Supplementary figure legends
