# Supplementary material for: Increased long noncoding RNA maternally expressed gene 3 contributes to podocyte injury induced by high glucose through regulation of mitochondrial fission
Source: Cell Death Dis. 2020 Sep 29;11(9):814. doi: 10.1038/s41419-020-03022-7 (PMC7525535; doi:10.1038/s41419-020-03022-7)
Supplement: Supplementary file 1 — Supplementary methods [file 41419_2020_3022_MOESM1_ESM.docx]

**Increased long noncoding RNA maternally expressed gene 3 contributes to podocyte injury induced by high glucose through regulation of mitochondrial fission**

Qiongxia Deng^1,2^, Ruowei Wen^1,2^, Sirui Liu^1,2^, Xiaoqiu Chen^1,2^ , Shicong Song^1,2^, Xuehong Li^1,2^, Zhongzhen Su^3^, Cheng Wang^1,2^

1. Division of nephrology, Department of medicine, the Fifth affiliated hospital Sun Yat-Sen University, Zhuhai, Guangdong, China, 519000
2. Guangdong Provincial Key Laboratory of Biomedical Imaging, the Fifth affiliated hospital Sun Yat-Sen University, Zhuhai, Guangdong, China, 519000
3. Department of Ultrasound, the Fifth affiliated hospital Sun Yat-Sen University, Zhuhai, Guangdong, China, 519000

Correspondence to

Zhongzhen Su Department of Ultrasound, the Fifth affiliated hospital Sun Yat-Sen University, Zhuhai, Guangdong, China, 519000

E-mail: sp9313@126.com

Cheng Wang

Division of nephrology, Department of medicine, the Fifth affiliated hospital Sun Yat-Sen University, Zhuhai, Guangdong, China, 519000

Guangdong Provincial Key Laboratory of Biomedical Imaging, the Fifth affiliated hospital Sun Yat-Sen University, Zhuhai, Guangdong, China, 519000

E-mail:wangch2@mail.sysu.edu.cn

Qiongxia Deng and Ruowei Wen contributed equally to this work.

Running title: podocyte mitochodrial fission

**Supplementary Materials and Methods**

**Genotyping**

At 3 weeks of age, mouse tail DNA was isolated using a Tissue Genomic DNA Extraction Kit (TIANGEN, Beijing) according to the manufacturer’s instructions. The primers used for PCR genotyping were as follows. Neo deletion PCR primers F1 (5’- GCATTACCACAGGGACCCCATTT -3’) and R1 (5’- CTGACGGACTATCTTGATACGGG -3’) produced 249 bp wild-type DNA or 420 bp floxed DNA. Puro deletion primers F2 (5’- CCCACTTCCTCATTCGCACCG -3’) and R2 (5’- GCTCTTGGACTCTCTCGGCTG -3’) produced 225 bp wild type Meg3 DNA or 357 bp floxed Meg3 DNA. NPHS2-Cre PCR primers F3 (5’- CGGTTATTCAACTTGCACCA -3’) and R3 (5’- GCGCTGCTGCTCCAG -3’) amplified 200 bp NPHS2-Cre recombinase. PCR was performed according to the PCR kit protocol, and the products were resolved on a 3% agarose gel using the TAE buffer system.

**Arraystar LncRNA Array**

A lncRNA array was performed under the support of Kanchen Biotech (200233; Shanghai, China). Briefly, differentiated human podocytes were treated with normal glucose (NG, 5.5 mmol/l) and high glucose (HG, 30 mmol/l) medium for 72 h. The cells were lysed and RNA was isolated using TRIzol reagent (Invitrogen). All experiments were performed in triplicate. Total RNA was amplified and labeled using the Quick Amp Labeling Kit, One-Color (Agilent Technologies, Santa Clara, CA, USA; 5190-0442) according to the manufacturer’s instructions. Labeled cRNA was purified using the RNeasy Mini Kit (Qiagen, Hilden, Germany; 74104) and hybridized with the Agilent Gene Expression Hybridization Kit (Agilent Technologies; 5188-5242). The microarray was scanned with Agilent Microarray Scanner (Agilent Technologies; G2565BA). Agilent Feature Extraction software (version 11.0.1.1) was used to analyze acquired array images. Quantile normalization and data processing were performed using the GeneSpring GX v12.1 software package (Agilent Technologies). Following quantile normalization of raw data, lncRNAs in which at least three of the six samples had flags in Present or Marginal (“All Targets Value”) were chosen for further analysis. Differentially expressed lncRNAs with statistical significance between the two groups were identified using P-value/FDR filtering. Differentially expressed lncRNAs between the two samples were identified through fold change filtering. Hierarchical clustering and combined analysis were performed using homemade scripts. This data have been deposited in NCBI's Gene Expression Omnibus (Edgar et al., 2002) and are accessible through GEO Series accession number GSE155188 (https://www.ncbi.nlm.nih.gov/geo/query/acc.cgi?acc=GSE155188).

**CRISPR/Cas9-mediated targeting**.

The double-nickase/CRISPR-Cas9 System was used to generate stable clones with knockout (KO) of Meg3 in cultured human podocytes. Briefly, two pairs of guide RNAs (gRNAs), targeting the Meg3 promoter region and exon 3 (gRNA1F/1R and gRNA2F/2R; see Supplemental Table S1 for the targeting sequence), were designed against sequences obtained from the human genome and cloned into PX459M (Addgene) to create the PX459M-gRNA1-gRNA2 multiplex system. All constructs were sequence validated before transient transfection into undifferentiated human podocytes using Lipofectamine 3000 (Thermo Fisher Scientific). Genomic DNA was extracted from puromycin-resistant cells and genotyping was performed. The selected cells were cultured as single clones and analyzed for Meg3 expression levels by qRT-PCR.

**Lentiviral transduction**

A stable lncRNA Meg3-OE human podocyte line was established using lentivirus infection. The target sequence was connected with lentivirus vector (pHBLV-CMV-MCS-3FLAG-EF1-ZsGreen-T2A-PURO, Hanbio Biotechnology, Shanghai, China) following amplification by PCR (h-Meg3-F, 5'- TACTAGAGGATCTATTTCCGGTGAATTCAGCCCCTAGCGCAGACGGCGG -3' and h-Meg3-R, 5'- AGTCACTTAAGCTTGGTACCGAGGATCCACATTGAAATGATTAGCC -3'). The vectors carrying the target sequence were transfected into undifferentiated human podocytes following amplification by 293T cells. Finally, the positive clones were selected using 1 μg/ml of puromycin and verified by real-time PCR and flow cytometry.

**Western blot analysis**

Cells were lysed in RIPA buffer (Beyotime Technology, Shanghai, China) containing 1 mM PMSF at 4°C. The suspension was centrifuged at 14,000 × g and the medium containing the proteins was collected. A total of 50 μg of protein lysate was diluted in loading buffer, separated by 10% SDS-PAGE (Bio-Rad Laboratories, Hercules, CA, USA), and transferred to a polyvinylidene fluoride membrane at 100 V for 1 h 30 min. The membrane was rinsed in Tris-buffered saline followed by rinsing in blocking buffer (5% milk powder) for 5 min. The membrane was immersed in blocking buffer for 1 h before incubation with the following primary antibodies overnight at 4˚C: anti-Nephrin (ab58968, Abcam, 1:1000); anti-Synaptopodin (sc-515842, Santa Cruz Biotechnology, 1:500); anti-Mitofusin1 (ab57602, Abcam, 1:1000); anti-Mitofusin2 (ab56889, Abcam, 1:5000); anti-Opa1 (ab42364, Abcam, 1:1000); anti-GAPDH (Cell Signaling Technology, 2118S, 1:100); anti-Drp1 (ab184247, Abcam, 1:1000); and anti-pDrp1 (S637) (ab193216, Abcam, 1:500). After rinsing in wash buffer, membranes were incubated with horseradish peroxidase-conjugated secondary antibody for 1 h at room temperature and developed using ECL reagent. Densitometric analyses were conducted using Image J software.

**Quantitative real-time PCR**

Total RNA was isolated from differentiated human podocytes and primary mouse podocytes using TRIzol reagent (TaKaRa Biotechnology, Dalian, China). The purity and concentration of RNA was measured using the Nano Drop 2000 (Thermo Fisher Scientific). cDNA was prepared using the PrimeScript RT Reagent Kit (TaKaRa Biotechnology, RR037A) according to the manufacturer’s instructions. qRT-PCR was performed using SYBR Green (TaKaRa Biotechnology, RR047A). The DNA oligos used are shown in Supplementary Table 1.

**Urine and serum analysis**

Urine microalbumin was measured using an ELISA kit (Exocell, Philadelphia, PA, USA), and the values were normalized with urine creatinine levels, which were quantified in the same samples with a urinary creatinine assay kit (Exocell) according to the manufacturer’s instructions. The urine albumin excretion rate was calculated as the urine albumin/creatinine ratio (UACR). Blood urea nitrogen was measured in serum using a Urea Nitrogen Colorimetric Detection Kit (Invitrogen) according to the manufacturer’s instructions.

**Histopathology Anaysis by light microscope and Transmission Electron Microscopy**

Mice were perfused with normal saline prior to kidney removal. Kidney tissues for light microscopy were immediately fixed in 4% paraformaldehyde overnight, embedded in paraffin, and further processed for hematoxylin and eosin, PAS, PASM, and Masson’s trichrome staining. Light microscope analyses were based on 30 randomly selected glomeruli per mouse in each group using Image J software. Mesangial expansion was defined as PAS-positive and nuclei-free area in the mesangium, while assessment of glomerulosclerosis was based on the area of collagen deposition detected by Masson’s trichrome staining. Tissues for electron microscopy were fixed in 2.5% glutaraldehyde (Alfa Aesar, Tewksbury, MA, USA). After washing, tissue blocks were dehydrated and embedded in Epon812 Resin (Ted Pella, Inc., Redding, CA, USA). Ultrathin sections (50–70 nm) were cut, stained with 2% uranyl acetate, and photographed using a 100 kV transmission electron microscope (Japan Electron Optics Laboratory Co., Ltd., JEM-1400 PLUS). ImageJ software was used to quantify the thickness of the glomerular basement membrane and evaluate the degree of foot process fusion and effacement under 12,000× magnification. Images at 30,000× magnification were used to measure alterations in mitochondrial morphology as previously described^1^. The morphology of 200 randomly selected mitochondrias were determined for each group.

**Immunofluorescence staining**

Kidney sections from each group were incubated with primary antibodies against Synaptopodin (1:100; Santa Cruz, sc-515842), Drp-1 (1:250; Abcam, ab184247), pDrp1 (S637) (1:500; Biobyt, Orb127984), and WT1 (1:50) (Abcam, ab89901) for 1 h at room temperature. The sections were subsequently washed with PBS and incubated with the corresponding fluorescence-linked secondary antibodies, Cy3 goat anti-mouse IgG, or FITC swine anti-rabbit IgG. After staining, the sections were counterstained with DAPI and visualized using confocal microscopy (Zeiss, Germany).

**References**

1. Galvan, D. L. et al. Drp1S600 phosphorylation regulates mitochondrial fission and progression of nephropathy in diabetic mice. *J. Clin. Invest*. **129**, 2807-2823 (2019).
