# Supplementary figures and images for: Increased long noncoding RNA maternally expressed gene 3 contributes to podocyte injury induced by high glucose through regulation of mitochondrial fission
Source: Cell Death Dis. 2020 Sep 29;11(9):814. doi: 10.1038/s41419-020-03022-7 (PMC7525535; doi:10.1038/s41419-020-03022-7)

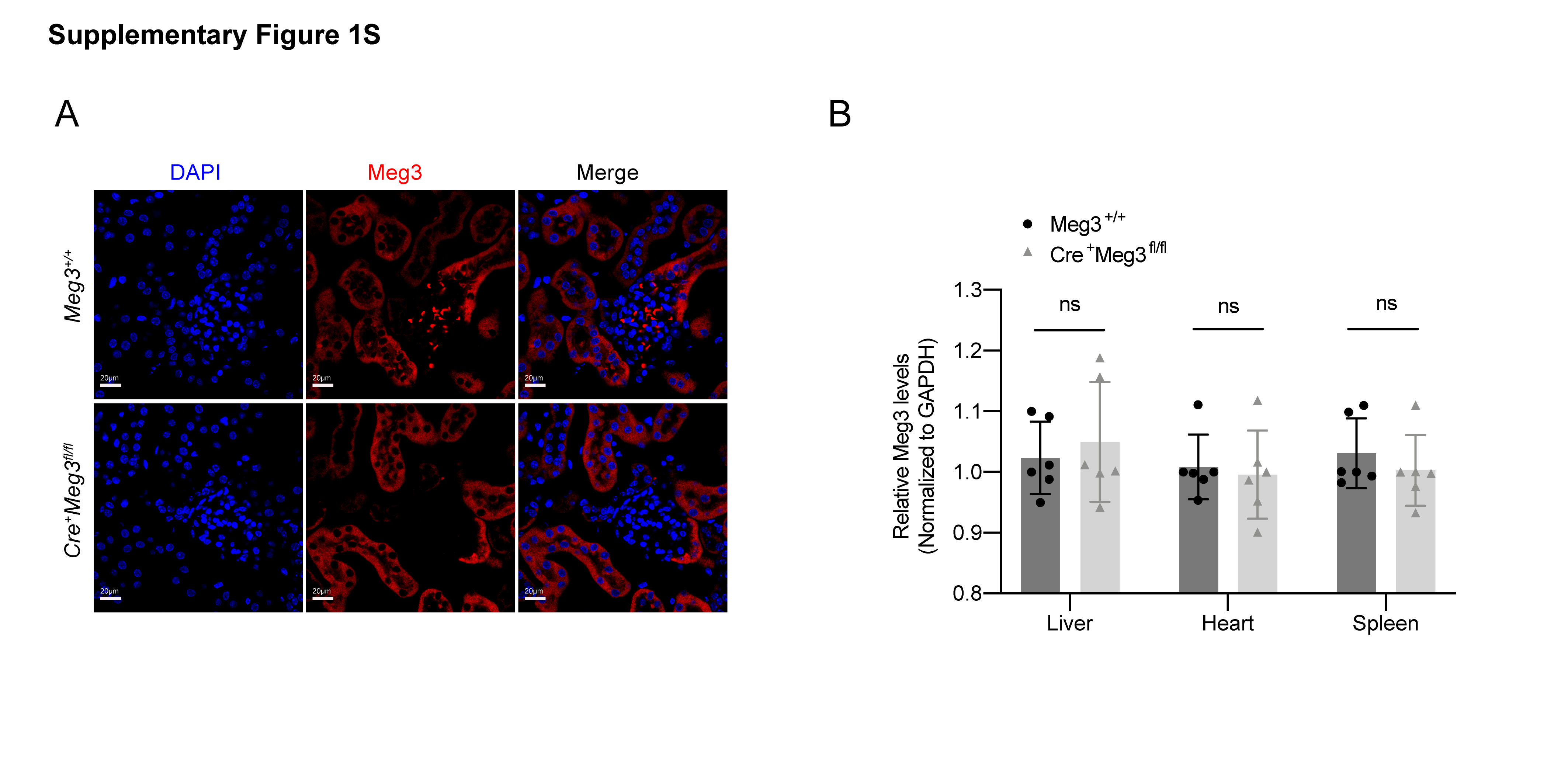

Supplement: Supplementary file 2 — Fig-1S [file 41419_2020_3022_MOESM2_ESM.tif]

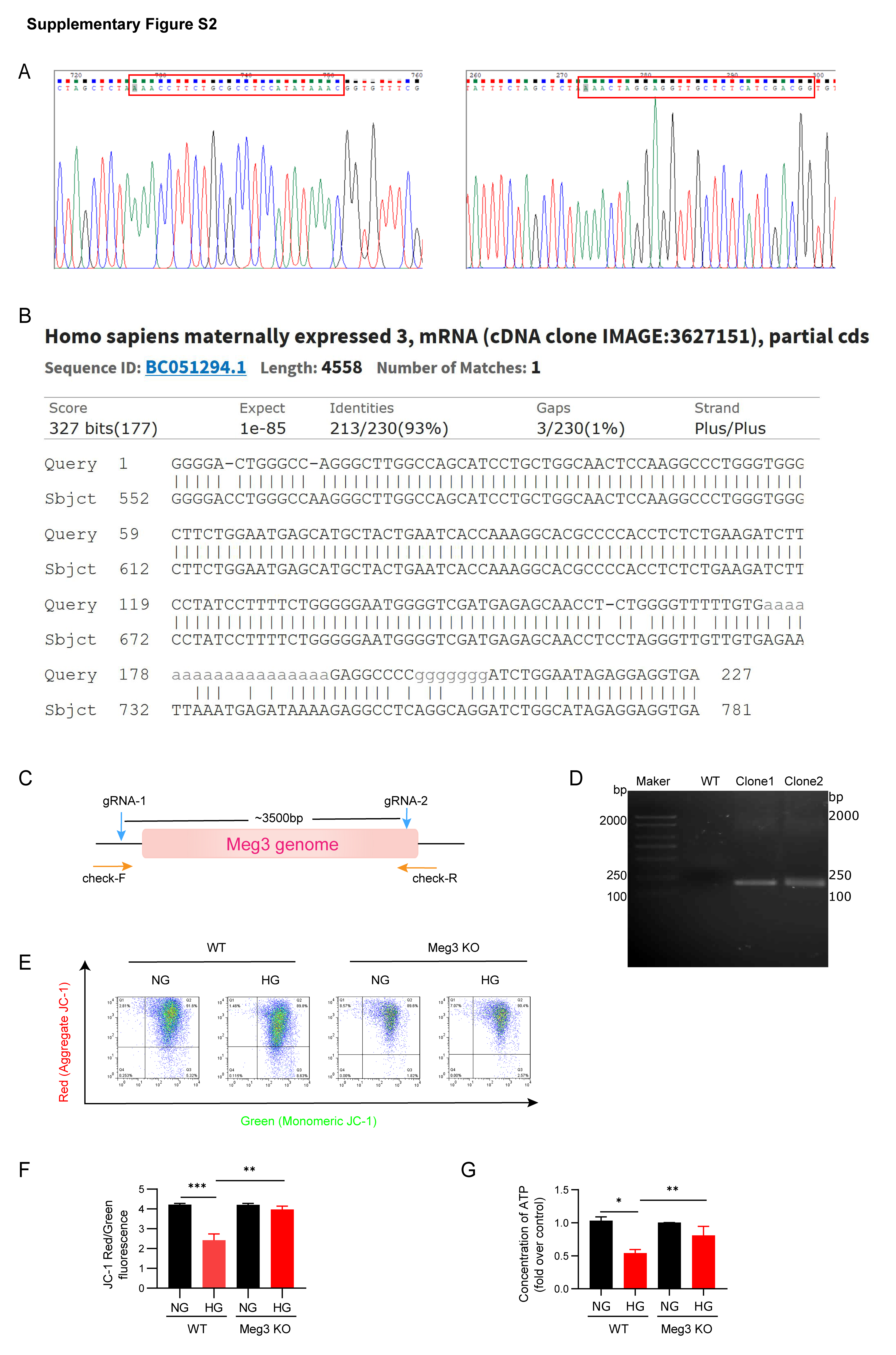

Supplement: Supplementary file 3 — Fig-2S [file 41419_2020_3022_MOESM3_ESM.tif]

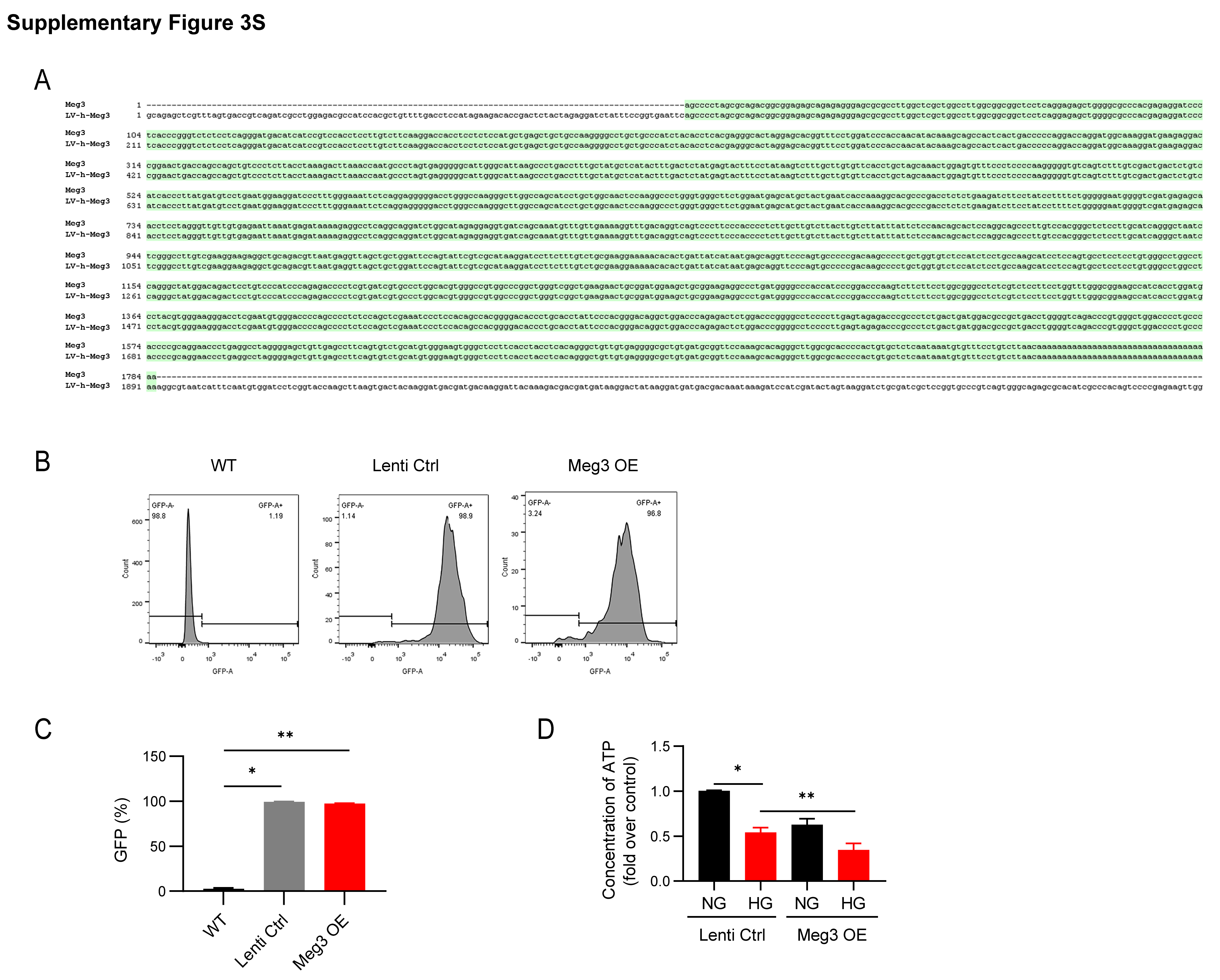

Supplement: Supplementary file 4 — Fig-3S [file 41419_2020_3022_MOESM4_ESM.tif]
