# Supplementary material for: Increased long noncoding RNA maternally expressed gene 3 contributes to podocyte injury induced by high glucose through regulation of mitochondrial fission
Source: Cell Death Dis. 2020 Sep 29;11(9):814. doi: 10.1038/s41419-020-03022-7 (PMC7525535; doi:10.1038/s41419-020-03022-7)
Supplement: Supplementary file 5 — Supplementary Table S1 [file 41419_2020_3022_MOESM5_ESM.docx]

| **Supplementary Table1. Sequences of primers used for plasmid construction and qRT-PCR.** | |
| --- | --- |
| **Primer names** | **Sequences** |
| h-Meg3 gRNA1 forward | 5'-CACCGTTTATATGGAGGCGCAGAAG-3' |
| h-Meg3 gRNA1 reverse | 5'- AAACCTTCTGCGCCTCCATATAAAC-3' |
| h-Meg3 gRNA2 forward | 5'- CACCGTCGATGAGAGCAACCTCCTA-3' |
| h-Meg3 gRNA2 reverse | 5'- AAACTAGGAGGTTGCTCTCATCGAC-3' |
| h-Meg3 check-gRNA forward | 5'-AATTTGTCATAGAATCTGGGGG-3' |
| h-Meg3 check-gRNA reverse | 5'-AAACATTTGCTGATCACCTCCT-3' |
| h-Meg3 lentiviral vector forward | 5'-TACTAGAGGATCTATTTCCGGTGAATTCAGCCCCTAGCGCAGACGGCGG-3' |
| h-Meg3 lentiviral vector reverse | 5'-AGTCACTTAAGCTTGGTACCGAGGATCCACATTGAAATGATTACGCC-3' |
| h-Meg3 forward | 5’-GCTATGCTCATACTTTGACTC-3’ |
| h-Meg3 reverse | 5’-CATCATAAGGGTGATGACAG-3' |
| h-GAPDH forward | 5’-AAGGCTGGGGCTCATTTGC-3’ |
| h-GAPDH reverse | 5’-GCTGATGATCTTGAGGCTGTTG-3’ |
| m-Meg3 forward | 5’- GGACTTCACGCACAACACG-3’ |
| m-Meg3 reverse | 5’-TTACAGTTGGAGGGTCCTGGG-3’ |
| m-GAPDH forward | 5’-TGCACCACCAACTGCTTAG-3’ |
| m-GAPDH reverse | 5’-GGATCAGGGATGATGTTC-3’ |
| m- Synaptopodin forward | 5’-TCCTCACCTAATGCCACACTC-3’ |
| m- Synaptopodin reverse | 5’-GCTGGAGGGTTTTGGTTGATA-3’ |
| m-Nephrin forward | 5’-GATGCGGAGTACGAGTGCC |
| m-Nephrin reverse | 5’-GGGGAACTAGGACGGAGAGG-3’ |
|  |  |
