# Supplementary material for: Increased long noncoding RNA maternally expressed gene 3 contributes to podocyte injury induced by high glucose through regulation of mitochondrial fission
Source: Cell Death Dis. 2020 Sep 29;11(9):814. doi: 10.1038/s41419-020-03022-7 (PMC7525535; doi:10.1038/s41419-020-03022-7)
Supplement: Supplementary file 6 — Supplementary figure legends [file 41419_2020_3022_MOESM6_ESM.docx]

**Supplementary Fig.1S The expression of LncRNA Meg3 in tubular cells and other tissue from *Meg3^+/+^* mice and *Cre^+^Meg3^fl/fl^* mice**

**A.** Representative images of confocal showing the expression of LncRNA Meg3 in tubular cells in *Meg3^+/+^* mice and *Cre^+^Meg3^fl/fl^* mice (Original magnification,×630, scale bar, 20μm). **B.** Comparsion of LncRNA Meg3 expression from liver, heart and spleen between *Meg3^+/+^* mice and *Cre^+^Meg3^fl/fl^* mice. Data were shown as mean ±SD in all statistical graphs. (n = 6 mice per group), ns, not significant.

**Supplementary Fig.2S** **Verification of the efficiency of CRISPR/Cas9 in Meg3 knockout and the regulation of Meg3 on mitochondrial bioenergetics.**

**A**. The sequencing results of PX459M-gRNA1-gRNA2 multiplex system. Circled in red were sequences of two pairs of gRNAs. **B**. Mutations of Meg3 Genome in Meg3 KO human podocytes.

**C**. The approximate positions of primers (check-F and check-R) used for genomic PCR. **D**. The results of identification of Meg3 KO clones by genomic PCR. **E**. Comparison of mitochondrial membrane potential in podocytes by flow cytometry analysis using JC-1. **F**. Quantitative analysis of mitochondrial membrane potential using JC-1. **G**. Relative ATP levels were assayed in human podocytes from different groups. Data were shown as mean ±SD in all statistical graphs. * indicated *P*< 0.05, ** indicated *P*< 0.01, ***indicated *P*< 0.001 (n = 3–10 each group).

**Supplementary Fig.3S Verification of the efficiency of lentiviral vectors that overexpress Meg3 and the regulation of Meg3 on mitochondrial bioenergetics.**

**A**. Comparison of sequencing of lentiviral vectors that overexpress Meg3 with original sequence of Meg3. The green area is the matching part with the target sequence. **B**. Flow cytometry and analysis of transfection efficiency of lentiviral vectors. **C**. Quantitative analysis of GFP levels of human podocytes from different groups. High level of GFP suggested high transfection efficiency. **D**. Relative ATP levels were assayed in human podocytes from different groups. Data were shown as mean ±SD. * indicated *P* < 0.05, ** indicated *P* < 0.01 (n = 3–10 each group).
